# Detection of *Bordetella pertussis* from Clinical Samples by Culture and End-Point PCR in Malaysian Patients

**DOI:** 10.1155/2013/324136

**Published:** 2013-05-07

**Authors:** Tan Xue Ting, Rohaidah Hashim, Norazah Ahmad, Khairul Hafizi Abdullah

**Affiliations:** Bacteriology Unit, Infectious Disease Research Centre, Institute for Medical Research, Jalan Pahang, 50588 Kuala Lumpur, Malaysia

## Abstract

Pertussis or whooping cough is a highly infectious respiratory disease caused by *Bordetella pertussis*. In vaccinating countries, infants, adolescents, and adults are relevant patients groups. A total of 707 clinical specimens were received from major hospitals in Malaysia in year 2011. These specimens were cultured on Regan-Lowe charcoal agar and subjected to end-point PCR, which amplified the repetitive insertion sequence *IS481* and pertussis toxin promoter gene. Out of these specimens, 275 were positive: 4 by culture only, 6 by both end-point PCR and culture, and 265 by end-point PCR only. The majority of the positive cases were from ≤3 months old patients (77.1%) (*P* < 0.001). There was no significant association between type of samples collected and end-point PCR results (*P* > 0.05). Our study showed that the end-point PCR technique was able to pick up more positive cases compared to culture method.

## 1. Introduction

Whooping cough is a major cause of the infant [1] and childhood mortality [2]. World Health Organization (WHO) reported about 16 million pertussis cases worldwide in 2008, in which 95% of cases occurred in developing countries and more than 100000 children died from this disease [1]. Pertussis still remains endemic despite the introduction of vaccination program in 1974 [3]. During 2003–2007, there were 43482 cases or an incidence of 4.1 per 100000 people reported from 20 European countries [4]. In the United States, the incidence of pertussis also increased from 3.53 in year 2007 to 5.54 in year 2009 [5]. Although pertussis is always classified as infants and children disease, an increasing number of cases in adolescents and adults group were also observed [2, 5]. 

According to the vaccination schedule of Malaysia, every citizen should be given vaccination at 2, 3, and 5 months old and a booster at 18 months old [6]. This is in line with the vaccination schedule recommended by WHO. Although there are two types of vaccines available, whole cell or acellular vaccine [6], the whole cell pertussis vaccine is the most widely used vaccine against pertussis [7]. The immunization coverage of three-dose pertussis vaccination is 95.3% [8]. Pertussis is a notifiable disease in Malaysia [9]. The incidence of pertussis has been less than 1 per 100000 people from 1989 to 2010 [10].

The clinical pertussis is defined by cough illness which last 2 weeks or more, with at least one of the symptoms of paroxysms of coughing, characteristic inspiratory whoop, and posttussive vomiting without apparent cause [11]. However, pertussis in adults and adolescents are rarely recognized because they normally have atypical course [12] or asymptomatic [13]. Hence, they become the reservoir of the disease and may transmit the infection to infants [2, 12]. Pertussis may also result in many complications such as pneumonia, apnoea, encephalopathy, death, loss of weight, convulsion, loss of bladder control, and rib fractures [14].

Culture was the gold standard for the diagnosis of pertussis due to its specificity; however, it is slow and not sensitive [15]. Detection by real-time polymerase chain reaction has been carried out by many laboratories and was found to be useful in the diagnosis of *B. pertussis* infection [16–18]. However, this method of detection requires the use of expensive instrument. Therefore, we applied end-point PCR technique and compared it with culture method to detect *B. pertussis* from suspected clinical samples. 

## 2. Materials and Methods

A total of 707 specimens were received from 57 Malaysian hospitals in year 2011. These samples were obtained from suspected pertussis cases as diagnosed by clinicians and sent to our laboratory for *B. pertussis* culture and end-point PCR detection. One sample was obtained per patient. The samples received were nasopharyngeal aspirates, nasopharyngeal swabs, tracheal aspirates, throat swabs, pernasal swabs, and endotracheal tube swabs. The nasopharyngeal aspirates were transported in sterile containers. The swab samples were transported in Regan-Lowe medium, Amies, Stuart, or without any transport medium. The samples were processed on the same day of arrival. 

### 2.1. Culture

All swab samples were rolled onto separate Regan-Lowe charcoal agar. Regan-Lowe charcoal agar plates were prepared from dehydrated powder obtained from Oxoid and 23 *µ*g mL^−1^ cephalexin, and 15% v/v defibrinated horse blood were added to the medium. For nasopharyngeal and tracheal aspirates, approximately 0.1 mL of the fluid was inoculated onto the Regan-Lowe charcoal agar plates. The agar plates were then incubated at 37°C aerobically. The plates were inspected after 72 hours. If no growth was observed, the plates were further reincubated until 120 hours. Any suspected colony was identified using *B. pertussis*-specific antisera (Difco, Sparks, MD, USA) [19].

### 2.2. Polymerase Chain Reaction

The same swab samples were transferred into 1.5 mL Eppendorf tubes containing phosphate buffered saline (PBS) (pH 7.4) and vortexed for 60 s to release cellular material into fluid. The swabs were then removed and the PBS suspensions were ready for DNA extraction. Nasal and tracheal aspirates were subjected to direct DNA extraction.

DNA extraction was performed using QIAamp DNA blood mini kit (QIAGEN), following the manufacturer's instructions. The DNA was kept at −20°C until further testing. The primers used for the end-point PCR are listed in Table 1. The DNA was first screened for the presence of *IS481* using BP1 and BP2 primers [19]. Amplification was performed using Biometra TPersonal version 2.0 (Biometra Gmbh, Goettingen, Germany) with the following parameters: predenaturation at 95°C for 5 min, followed by 30 cycles of denaturation at 95°C for 1 min, annealing at 57°C for 1 min, extension at 72°C for 1 min with a final extension at 72°C for 5 min. The expected end-point PCR product size for fragment of *IS481* is 151 bp.

If the screening test was positive, the extracted DNA was then subjected to end-point PCR amplification of pertussis toxin (PT) promoter using BPTOX F and BPTOX R primers [19]. The parameters used were as follows: predenaturation at 95°C for 5 min, followed by 30 cycles of denaturation at 95°C for 1 min, annealing at 60°C for 1 min, extension at 72°C at 1 min with a final extension at 72°C for 5 min. The expected end-point PCR product size of fragment of PT promoter is 190 bp.

The internal control primers Hg1 and Hg2 were used in both screening and confirmatory test to target a region of human hemoglobin gene as described by [20]. The expected end-point PCR product size for internal control is 268 bp.

The end-point PCR products were visualized by 1.5% agarose gel electrophoresis stained with RedSafe TM Nucleic Acid Staining Solution (iNtRON Biotechnology Inc., Sungnam, Kyungki-Do, Republic of Korea) and documented with a ChemiDoc XRS+ system (Bio-Rad, Hercules, CA, USA).

The end-point PCR result was considered positive if both target and internal control bands or target band alone was obtained. However, it was considered negative when the internal control band alone was observed. Cases were defined as positive if both screening and confirmatory tests were positive.

## 3. Results

From the 707 samples processed, 275 samples were positive for *Bordetella pertussis*. Four cases were detected by culture method alone, 6 were detected by both end-point PCR and culture methods, and another 265 were detected by end-point PCR alone. Overall, 454 samples were positive in *IS481* screening test and only 271 samples (60%) were positive in PT promoter confirmatory test. For those negative in confirmatory test (183 samples), the cases were recognized as *Bordetella* spp. 

Most of the positive cases were obtained from nasopharyngeal aspirates (40.9%), followed by nasopharyngeal swab (35.6%), pernasal swab (33.3%), tracheal aspirate (28.6%), and throat swab (14.3%) and none from endotracheal tube swab (Table 2). 

The majority of positive cases were from Kedah (42/275), Terengganu (39/275), and Selangor (35/275). The lowest number of pertussis cases was found in Kuala Lumpur with 2 cases only.

Positive cases were highest among patients aged ≤3 months old (212/275) followed by patients with aged 4–12 months old (37/275). However, there were also 12 cases detected from individuals between 13 to 24 months old and 14 cases in individuals more than 24 months old (Table 3).

## 4. Discussion

The end-point PCR assay has been used to detect *B. pertussis* in the routine work [19]. It was shown to be rapid and highly sensitive compared to culture which is slow and has low sensitivity. Although culture has high specificity, it can be affected by several factors such as specimen transport condition, illness duration, and antibiotic treatment [21, 22]. 

In this study, we screened the samples for the presence of *IS481* and proceeded to the detection of pertussis toxin when *IS481* screening was positive. The repetitive insertion sequence *IS481* was shown to be present in *B. pertussis* with 200 copy numbers [23, 24]. However, it was not specific enough as it is also present in *B. holmesii *and* B. bronchiseptica* [25]. The other major virulent determinant is pertussis toxin promoter region which is present as a single-copy gene. Single-target testing using* IS481* may miss up to 20% of true positive cases [17]. Therefore, we used dual-target PCR assay targeting *IS481* and pertussis toxin promoter region as practiced by other researchers to increase the sensitivity of the test [19, 26]. The internal control was introduced into the assay to differentiate between false negative and true negative results [27].

There were four cases that were culture positive but undetectable by our end-point PCR assay. We believe this could be due to the inhibitory substances which may be present in the specimens as described by Douglas et al. [28]. Mattoo and Cherry [29] have suggested treating the specimens with mucolytic agent to remove the inhibitory substances prior to the end-point PCR.

There were several factors that could lead to the culture sensitivity in this study. Many of the samples were not received on the same day of specimen collection except from the states of Selangor, Kuala Lumpur, and Putrajaya, which are geographically near our laboratory. Besides, we found that some of the swabs were not transported in the proper transport media, namely, Regan-Lowe medium, as suggested by WHO [30]. Prior antibiotic treatment before collection of samples could also affect the growth of *B. pertussis* [31, 32]. Most of the patients had been on antibiotic treatment before their samples were collected and this could contribute to the inability to recover viable bacteria by culture method.

There was no significant association between the types of samples collected and end-point PCR results (*P* > 0.05) (chi-square test by software SPSS 16). However, nasopharyngeal aspirate samples had the highest number of positive end-point PCR results (40.3%) compared to nasopharyngeal swab (35.6%) and others (21.6%). Douglas et al. [28] described that the low detection in the swab specimens was because of the insufficient cells found on the swabs. In contrast, Farrell et al. [23] obtained high percentage of end-point PCR positive from throat swab specimens in their study. The detection from throat swab in our study was low, that is, 0.4% and 10% from end-point PCR and culture, respectively. The small number of throat swab samples in this study could also contribute to the low number of positive results.

In our study, infant aged ≤3 months old was the highest group to be infected by *B. pertussis*. This result was expected because they have not been fully vaccinated. The proportion of those in the age group ≤3 months old (45.3%) with positive result was significantly higher than that in the age group >3 months old (26.4%) (*P* < 0.001) (chi-square test by software SPSS 16). This could be due to the direct transmission from their parents, relatives, or guardians who may be the reservoir for the bacteria. This idea was supported by Kenneth et al. [33] who stated that immunity against pertussis vaccination will wane after 10-year-vaccination, and the transmission rate to infant by household caretaker ranged between 30–40%. Hence, booster immunizations of adolescents in 10–19 years old are strongly suggested to reduce disease burden, healthcare setting cost and increase work productivity [33, 34]. 

## 5. Conclusion

In conclusion, our study showed that more pertussis cases were detected by end-point PCR compared with culture method. Most of the positive samples were from nasopharyngeal aspirates. However, there is no significant association between the type of specimens and the end-point PCR results. Most of the cases involved ≤3 months old patients who had not completed the scheduled vaccinations. Education and awareness are necessary to prevent the disease becoming widespread. 

## Figures and Tables

**Table 1:** Sequences of the primers.

Primer	Sequence
BP1	5′-GAT TCA ATA GGT TGT ATG CAT GGT T-3′
BP2	5′-TGG ACC ATT TCG AGT CGA CG-3′
BPTOX F	5′-GCG CAT GCG TGC AGA TTC GTC-3′
BPTOX R	5′-CCC TCT GCG TTT TGA TGG TGC C-3′
HG1	5′-CAA CTT CAT CCA CGT TCA CC-3′
HG2	5′-GAA GAG CCA AGG ACA GGT AC-3′

**Table 2:** Number of positive cases which detected from different specimens.

Specimen type	Total number of specimens	Number of positive specimens		
		Culture only	End-point PCR only	Culture and end-point PCR
Nasopharyngeal aspirate	521	3	207	3
Nasopharyngeal swab	149	0	50	3
Tracheal aspirate	7	0	2	0
Throat swab	14	1	1	0
Pernasal swab	15	0	5	0
Endotracheal tube swab	1	0	0	0

**Table 3:** Age range (month) of positive pertussis cases.

Age (month)	Total number of specimens	Number of pertussis cases		
		Culture only	End-point PCR only	Culture and end- point PCR
<1	37	0	13	0
1–3	431	4	189	6
4–6	105	0	20	0
7–9	38	0	7	0
10–12	32	0	10	0
13–15	7	0	4	0
16–18	9	0	3	0
19–21	7	0	1	0
22–24	8	0	4	0
>24	33	0	14	0
